# *Nanomaterials* 2021 Best Paper Awards: Announcement and Interview with the Winner

**DOI:** 10.3390/nano11123318

**Published:** 2021-12-07

**Authors:** Nanomaterials Editorial Office

**Affiliations:** MDPI AG, St. Alban-Anlage 66, 4052 Basel, Switzerland; nanomaterials@mdpi.com

After an extensive voting period, we are proud to present the Best Paper Award to “Carbon Dots and Graphene Quantum Dots in Electrochemical Biosensing [1]” from corresponding authors Dr. Susana Campuzano and Prof. Dr. José M. Pingarrón.

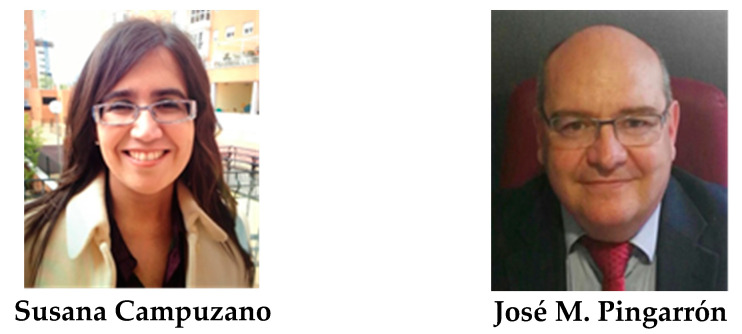



Susana Campuzano (Orcid 0000-0002-9928-6613) received her PhD in analytical chemistry from the Universidad Complutense de Madrid in 2004. Since 2005, she has worked as Assistant Professor at the Analytical Chemistry Department of the Chemistry Faculty of the same university, where she is currently head of the Electroanalysis and Electrochemical (Bio)Sensors (GEBE) research group. During 2008 and 2009, she enjoyed a Juan de la Cierva research contract in the Department of Molecular Microbiology and Infection Biology at the Centro de Investigaciones Biológicas Margarita Salas of the Consejo Superior de Investigaciones Científicas (CIB Margarita Salas-CSIC) in Madrid under the supervision of Prof. Ernesto García. She worked as a Research Scholar in the research group of Prof. Joseph Wang of the Department of Nanoengineering at the University of California San Diego (UCSD, USA) from January 2010 to July 2011. Her areas of interest include the development of affinity-based electrochemical bioplatforms with potential for multiplexed and/or multi-omics determinations in clinical and food safety. She has authored over 242 peer-reviewed papers (cited 7639 times; h index: 44, Scopus), 8 patents and 1 utility model, 20 book chapters and more than 220 contributions to national and international conferences. She is Associate Editor of the Electroanalysis Journal (Wiley-VCH) and an advisory board member of Analytical and Bioanalytical Chemistry (Springer), Scientific Reports (NPG) and Biosensors and Bioelectronics: X (Elsevier). She has participated both as a co-principal investigator and researcher in many national, regional and two European Projects and in contracts with private companies to implement laboratory developments in different fields.

José M. Pingarrón (Orcid 0000-0003-2271-1383) received his PhD in Chemical Sciences (Analytical Chemistry) in 1981 from the Universidad Complutense de Madrid (Spain). Postdoctoral stay at l’ École Nationale Supérieure de Chimie de Paris (1982-83). Visiting Professor at Cornell University, USA (1997). Professor of Analytical Chemistry at UCM since 1994. Head until November 2020 and currently member of the group “Electroanalysis and Electrochemical (Bio)sensors” at Complutense University of Madrid. Author or co-author of 471 published papers; 67% in Q1; 28% in D1; h-index 66/56 (Google Scholar/Scopus); total citations 18686/14492 (Google Scholar/Scopus), 35 book chapters, 2 textbooks, 12 patents and 31 Doctoral Theses supervised. Editor for Europe until 2019 and currently Senior Honorary Advisor of the journal Electroanalysis (Wiley-VCH). Principal Investigator of regional, national, international competitive projects and collaboration with companies. President of the Spanish Society of Analytical Chemistry (1998–2001). Vice-President of the Spanish Royal Society of Chemistry (2015–2019). Member of the Board of Directors of the Confederation of Scientific Societies of Spain (2014–2018). Medal of the Faculty of Chemistry. Research Award in Analytical Chemistry of the Royal Spanish Society of Chemistry (RSEQ) (2012). Fellow of the International Society of Electrochemistry (2017). Scientific Research Award 2018 of the Electrochemistry Group of the RSEQ. Medal of Honor of the Faculty of Nursing, Physiotherapy and Podiatry of the Complutense University of Madrid (2019). Member of the management team of the Basic Chemistry subprogram of the National Research Plan of the Ministry of Economy and Innovation (2008–2015). President of the Advisory Council for Science, Technology, and Innovation, 2017–2018. Vice-rector of Knowledge Transfer and Entrepreneurship (UCM) 2015–2018. President of the OTRI Network, 2015–2016. Executive Secretary of the R+D+I Sectorial of CRUE, 2016–2018. Currently he holds the position of Secretary General of Universities in the Spanish Government.

On behalf of the *Nanomaterials* editorial office staff and award evaluation committee, we congratulate Dr. Susana Campuzano and Prof. Dr. José M. Pingarrón on their excellent performance and wish them all the best for their future career.

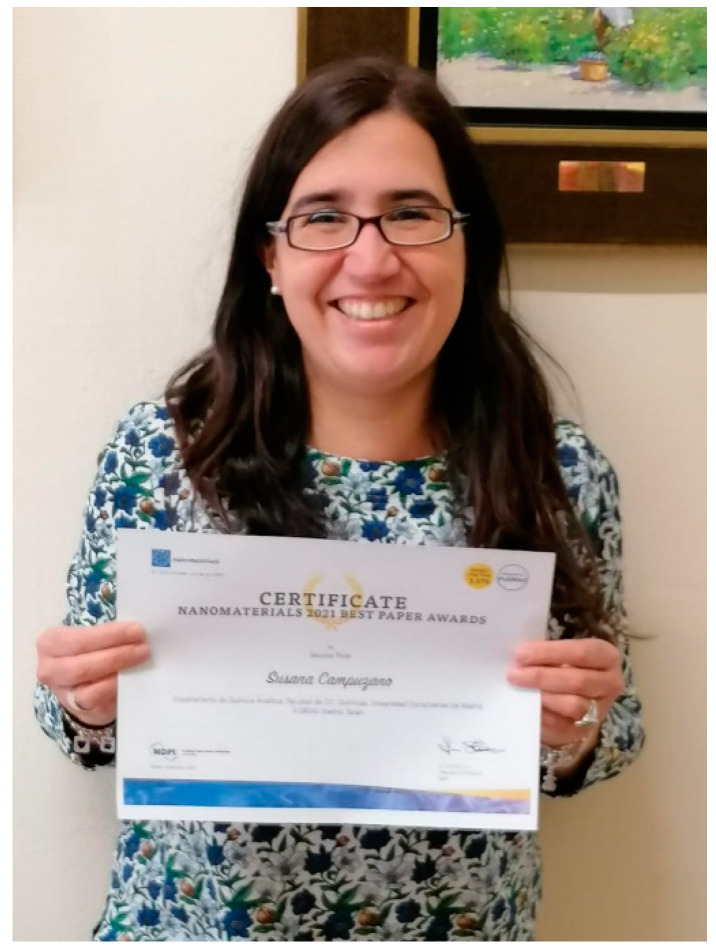

**Interview with the Winner**

*1.*  *Could you briefly introduce yourself and share the process of writing this article with the readers?*

The two corresponding authors of this review are Associate Professor (Susana Campuzano) and Full Professor (José M. Pingarrón) of the Department of Analytical Chemistry of the Faculty of Chemistry of the Complutense University of Madrid (Spain). Prof. Pingarrón was the mentor of the research group “Electroanalysis and (Bio)Electrochemical Sensors” (GEBE) until November 2020, and Prof. Susana Campuzano took over leadership from then. 

Regarding the writing of this paper, we must indicate that it was prepared in response to an invitation we received from our collaborators Prof. Dr. Dimitrios P. Nikolelis and Dr. Georgia-Paraskevi Nikoleli, as Guest Editors of this Journal’s Special Issue devoted to “Nanostructured Biosensors”. Our contribution was very well rated by the four expert referees who judged it, who expressed very positive and helpful comments. Moreover, its preparation, consideration and publication by this Journal was a pleasant and efficient process in which we felt at all times supported by the kind Editorial Team of *Nanomaterials*, in this particular case, by Ms. Alisa Zhai and Ms. Mani Wang (Assistant Editors).

*2.*  *What are you currently researching and why did you choose this research field?*

Our current areas of interest include the development of affinity-based electrochemical bioplatforms with potential for multiplexed and/or multi-omics determinations in clinical and food safety.

*3.*  *Which research topics do you think will be of particular interest to the research community in the coming years?*

There are so many. Focusing on what is closest to us, we would say the development of novel biotools able to assist in personalized precision medicine both of known diseases in our society and those, like COVID-19, which may arise unexpectedly, turning our lives upside down, to help us feel safe against potential environmental or food risks. Naturally, the development of these devices in suitable formats (portable, user-friendly and/or wearable) so that they incur as quickly as possible in our daily routines to ensure our well-being against known, unknown and unexpected threats.

*4.*  *As a young researcher, what was the biggest challenge you encountered in your research journey? How did you solve it?*

The great dedication that this work requires and the tremendous competition. Putting desire and passion in what we do and, of course, the support of a great research team and collaboration with dedicated people who love their work as much as we do is indispensable.

*5.*  *Can you briefly describe the key to a happy laboratory life?*

We think it is essential to be able to work in a team and in an optimal environment, in which everyone strives to improve personally and to value and get the best out of those around them. And, of course, not to fall into monotony and to continue training, learning and enjoying new things every day.

*6.*  *If you have the opportunity, will you actively apply to attend academic conferences? What do you think you can learn from participating in conferences that is different from working in a lab?*

Indeed, we are very active in conference participation and in the internalization of our research. In addition to learn new scientific advances, we believe that active participation in congresses and conferences is crucial to keep up to date and consolidate a network of national and international contacts that can be used by younger researchers to carry out research stays in different centers and groups, which is beneficial for their personal and professional maturity, and essential to establish one-time or ongoing collaborations over time or apply jointly to competitive international calls.

*7.*  *Which qualities do you think young researchers need?*

Passion, perseverance, desire, imagination, humility, spirit of improvement and patience.

*8.*  *As an open access journal. How do you think open access impacts the authors?*

Open access is definitely a catalyst to put our small achievements at the service of the entire scientific community and our society, which has been proven over the last two years, for example, by developing vaccines or new tools to detect COVID-19 infection in record time, to be the best weapon to be able to respond jointly and effectively to what the future holds for us.
